# A Review of Nuclear Medicine Approaches in the Diagnosis and the Treatment of Gynecological Malignancies

**DOI:** 10.3390/cancers14071779

**Published:** 2022-03-31

**Authors:** Nasim Vahidfar, Saeed Farzanefar, Hojjat Ahmadzadehfar, Eóin N. Molloy, Elisabeth Eppard

**Affiliations:** 1 Department of Nuclear Medicine, Vali-Asr Hospital, Tehran University of Medical Sciences, Tehran 1419733133, Iran; n-vahidfar@sina.tums.ac.ir (N.V.); farzanehfar@tums.ac.ir (S.F.); 2 Department of Nuclear Medicine, Klinikum Westfalen, 44309 Dortmund, Germany; hojjat.ahmadzadehfar@ruhr-uni-bochum.de; 3 University Clinic for Radiology and Nuclear Medicine, Faculty of Medicine, Otto von Guericke University (OvGU), 39120 Magdeburg, Germany; eoin.molloy@med.ovgu.de; 4 German Center for Neurodegenerative Diseases (DZNE), 39120 Magdeburg, Germany

**Keywords:** gynecology, radiopharmaceutical, positron emission tomography/computer tomography (PET/CT), single-photon emission computed tomography/computed tomography (SPECT/CT)

## Abstract

**Simple Summary:**

In this literature review, we will provide a short overview of the role of nuclear medicine in the diagnosis of obstetric and gynecological cancers.

**Abstract:**

Nuclear medicine is defined as the diagnosis and the treatment of disease using radiolabeled compounds known as radiopharmaceuticals. Single-photon emission computed tomography/computed tomography (SPECT/CT) and positron emission tomography/computer tomography (PET/CT) based radiopharmaceuticals have proven reliable in diagnostic imaging in nuclear medicine and cancer treatment. One of the most critical cancers that also relies on an early diagnosis is gynecological cancer. Given that approximately 25% of all cancers in developing countries are a subset of gynecological cancer, investigating this cancer subtype is of significant clinical worth, particularly in light of its high rate of mortality. With accurate identification of high grade distant abdominal endometrial cancer as well as extra abdominal metastases, 18F-Fluorodeoxyglucose ([^18^F]FDG) PET/CT imaging is considered a valuable step forward in the investigation of gynecological cancer. Considering these factors, [^18^F]FDG PET/CT imaging can assist in making management of patient therapy more feasible. In this literature review, we will provide a short overview of the role of nuclear medicine in the diagnosis of obstetric and gynecological cancers.

## 1. Introduction

Gynecological malignancies include ovarian, cervical, and endometrial cancer, and greatly affect female health and quality of life worldwide [1]. Despite promising advancements in the detection and the treatment of cancers, there are still uncertainties in the diagnostic methods, which in turn can contribute to patient mortality. Epidemiological population-based data from 1990 until 2019 have shown that breast cancer is the most frequent type of cancer in females, followed by cervical cancer, and then ovarian and uterine cancers [2,3]. Nuclear medicine based diagnostic imaging has played a considerable role in many aspects of the management of treatment planning, such as in predicting and staging the malignancies and in patient responses to treatment [4]. Several radiopharmaceuticals have been developed for diagnostic investigations of gynecological cancers, with encouraging results. For example, [^18^F]-fluorodeoxyglucose ([^18^F]FDG) is a promising radiopharmaceutical with extensive application in oncology [5]. Compared to benign tissues with lower rates of glycolysis and other normal tissues, [^18^F]FDG preferentially accumulates in malignant neoplasm with high rates of glycolytic activity [6,7].

Given that there is a significant increase in protein synthesis in these malignancies [8], following in-vivo amino acid consumption during protein synthesis could be very informative. It is hypothesized that the amino acid methionine plays a critical role as a primer member of proteins [9]. Carbon-11 radiolabeled methionine ([^11^C]C-MET) has been shown to be potentially useful as a positron emission tomography/computed tomography (PET/CT) tracer for imaging of various cancers [10,11,12,13,14]. Evidence suggests a possibly favorable effect of [^11^C]C-MET over [^18^F]FDG. For example, in a study on prostate cancer, [^11^C]C-MET more clearly detected lesions when compared to [^18^F]FDG [15]. Another advantage of [^11^C]C-MET over [^18^F]FDG is the lack of concentration of the former in infections and inflammations since protein synthesis does not take place in the above mentioned conditions compared to glucose metabolism [16], the primary target of [^18^F]FDG imaging. Additionally, it is also hypothesized that [^11^C]C-MET would show greater specificity in detecting gynecological malignancies [16]. The full potential for [^11^C]C-MET in imaging gynecological and obstetric malignancy, however, remains to be empirically verified. Further studies have also shown that 1-(2-hydroxy-3-[^18^F]fluoropropyl)-2-nitroimidazole ([^18^F]FMISO), a tumor hypoxia PET/CT tracer, could be a potent prognostic radiopharmaceutical for the evaluation of pre-therapeutic oxygen status in gynecological cancer [10].

**Figure 1:**
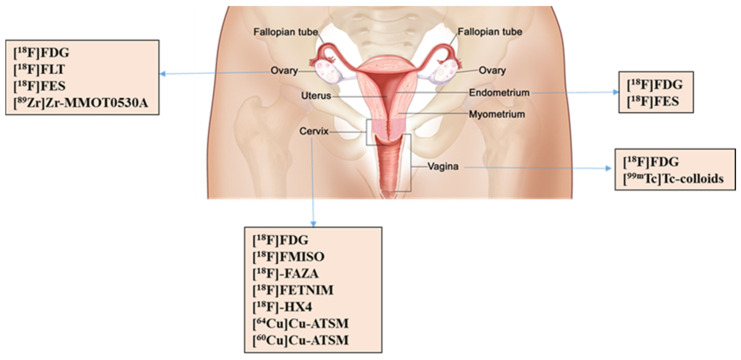
List of commonly applied radiopharmaceuticals in gynecology. [^18^F]FLT = [^18^F]Fluorothymidine, [^18^F]FES = 16a-[^18^F]-fluoro-17b-estradiol, [^89^Zr]Zr-MMOT0530A = ^89^Zr-labeled Monoclonal Antibody, [^18^F]FAZA = [^18^F]Fluoroazomycin-arabinoside, ([^18^F]FETNIM = [^18^F]Fluoroerythronitroimidazole, [^18^F]HX4 = [^18^F]Flortanidazole [^64^Cu]Cu-ATSM = [^64^Cu]Cu-Diacetyl-bis(N4-methylthiosemicarbazone), [^60^Cu]Cu-ATSM = [^60^Cu]Cu-Diacetyl-bis(N4-methylthiosemicarbazone), ^99m^Tc-colloids = ^99m^Tc-Sulfur Colloid.

Substantial progress has been made in the field of nuclear medicine in relation to gynecological neoplasms, which are one of the most common and fatal cancers worldwide [17]. Staging of these cancers is strongly dependent on the successful evaluation of primary lymph node (LN) status in the determination of distant metastases [17]. Notably, numerous technetium-99m (^99m^Tc) based colloidal radiopharmaceuticals have been developed and successfully applied for lymphoscintigraphy of gynecological cancers [18]. The most critical gynecological neoplasms include cervix uteri neoplasms and ovarian cancers, followed by endometrial cancers [19]. Less serious are vulvar and vaginal cancer, as well as localized melanomas in the female reproductive system, which typically lead to mortality in rare cases only [19]. In this review article, we aim to summarize the diagnostic progress in nuclear medicine in the context of gynecological cancers (Figure 1). We further discuss hypoxia radiopharmaceuticals, which play a key role as monitoring tracers for gynecological cancers and the fact that, despite greater availability of single-photon emission computed tomography/computed tomography (SPECT/CT) compared to PET/CT, more potential PET tracers have been used in clinical trials for gynecological abnormalities that are discussed in the following review.

## 2. Cervical Cancer

**Figure 2:**
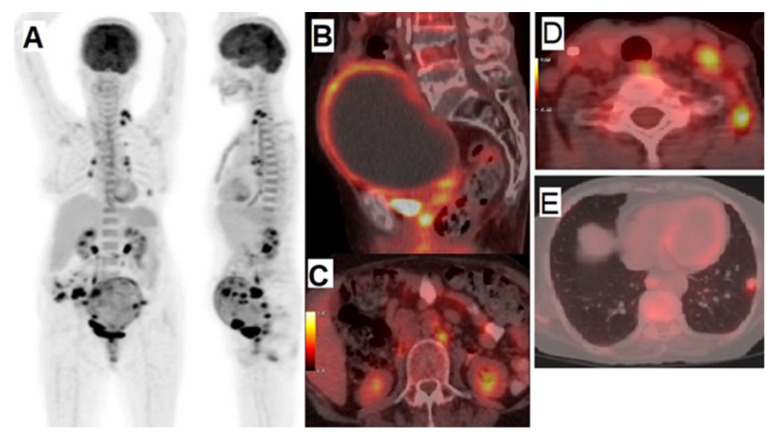
A 66-year-old female presented with a pelvic mass and vaginal bleeding. Cervical squamous cell carcinoma (SCC) was confirmed via biopsy and referred for staging. (**A**) Disease distribution visualized with [^18^F]FDG PET/CT imaging. (**B**) Sagittal pelvis image showing primary tumor leading to hydrometra and regional lymphadenopathies. (**C**) Retroperitoneal and (**D**) Supraclavicular lymph node metastases, in addition to lung metastases (**E**). (Pictures with courtesy from Department of Nuclear Medicine, Vali-Asr Hospital, Tehran University of Medical Sciences, Tehran, Iran).

Globally, cervical cancer is the second most serious gynecological malignancy in terms of mortality in patients under 35 years of age [20,21]. As a result, numerous studies have been conducted in order to understand its epidemiology and possible etiology [22,23,24]. A main consideration in the management of cervical cancer is the appropriate staging of access to effective treatment methods and patients’ prognosis [21]. One such consideration is that the detection resolution of PET/CT for staging of primary tumors of cervical cancers is limited [21]. Consequently, the use of MRI for imaging tumor volume, size, and the extent of parametrial invasion may be superior, acting as a gold standard for evaluating the locoregional extension of cervical cancer [21,25]. Nevertheless, [^18^F]FDG PET, provides metabolic information by depicting glycolytic tumor activity, and it can also obtain additional information in the staging of primary cervical cancers [25]. Pawar et al. assessed the success rate of PET/CT in a retrospective study of 56 patients with gynecological malignancy including cervix carcinomas (23 patients). It was shown that PET/CT offers a high diagnostic accuracy, both in the evaluation of suspected tumor recurrence and in persistent disease [26]. The authors concluded that PET/CT has particular value in primary cervical cancer, which is related to the diagnosis of extra-pelvic abnormalities, the detection of recurrence, and the monitoring of patients after treatment [26]. In another retrospective analysis of the accuracy of [^18^F]FDG PET/CT, the rate of success in the initial stages of cervical tumors was estimated to be 100% [27]. Further studies and clinical observations have demonstrated that the combined PET/CT has greater accuracy compared to PET imaging alone [28,29]. Generally, it has been concluded that [^18^F]FDG PET/CT is a choice modality for investigations of pretreatment staging and post-treatment surveillance of cervical cancer (Figure 2) [30]. One of the most considerable and adverse criteria of cervical cancer is tumor hypoxia [31,32]. Hypoxia is defined as oxygen insufficiency in cells, and it can be used as a prognostic indicator. Hypoxia has shown particular utility in therapeutic cancer management, including responses to chemotherapy or radiation therapy [33,34,35,36]. Another noteworthy aspect of hypoxia is the prediction of metastases in tumor cells which are related to hypoxia’s role in deoxyribonucleic acid (DNA) mutations and malignant, atypical cells [10]. Given these observations, the evaluation of hypoxia in treatment management is essential, particularly for locally advanced stages and local recurrences, which occur more than expected in cervical cancer [37].

### Hypoxia Imaging Radiopharmaceuticals for Cervical Cancer

The two major categories of radiolabeled hypoxia imaging agents are nitroimidazole derivatives and diacetyl-bis(N4-methylthiosemicarbazone) (ATSM) analogues [38]. Among the nitroimidazoles, [^18^F]FMISO has been widely used in recent clinical trials; [^18^F]FMISO can identify hypoxic tumor sub-volumes and track their spatio-temporal dynamics [39,40]. In one study, sixteen patients with histopathologically verified and locally advanced cervical cancer underwent [^18^F]FDG/[^18^F]FMISO PET/magnetic resonance imaging (MRI) scans [41]. Results indicated that a [^18^F]FDG/[^18^F]FMISO PET/MRI scan is feasible in cervical cancer cases, providing complementary information about tumor biology and heterogeneity while identifying hypoxic tumor sub-volumes which are resistant to treatment methods [41]. All detected lesions involved in the hypoxic condition were identified, as was a strong functional correlation between [^18^F]FDG and [^18^F]FMISO [42]. Two other hypoxia imaging agents with variable pharmacokinetics compared to [^18^F]FMISO [38] are [^18^F]F-fluoroazomycin-arabinoside ([^18^F]FAZA) and [^18^F]F-fluoroerythronitroimidazole ([^18^F]FETNIM); and, [^18^F]FETNIM has shown lower tumor to non-target accumulation in lung cancer patients [43]. Since lipophilicity of [^18^F]FMISO is high, it can pass cell membranes through passive diffusion. However, this passive diffusion causes a slow clearance and also a poor tumor to normal tissue ratio [44]. A less lipophilic hypoxia agent ([^18^F]FAZA) has also been evaluated in clinical trials. A comparison of [^18^F]FMISO and [^18^F]FAZA demonstrates extensive functional correlations, suggesting its comparable utility in the detection and the specification of hypoxic tumor volumes using PET/CT [45]. In a pilot study of uterine cervix cancer patients undergoing MRI guided adaptive radiotherapy, it was further demonstrated that [^18^F]FAZA PET/CT for the detection of radio-resistance related to hypoxia is practical [46], compared to morphologic repetitive MRI. However, according to clinical results in non-small-cell lung carcinoma (NSCLC), [^18^F]FMISO may be superior to [^18^F]FAZA, and it remains the gold standard for volume detection of tumor hypoxia [45]. This result was supported by a simulation study which introduced a new hypoxia agent named [^18^F]F-flortanidazole ([^18^F]HX4) [47]. According to the results, [^18^F]HX4—the third generation of hypoxia imaging agents—showed the fastest clearance rate, highest image contrast, and lowest background signals compared to [^18^F]FMISO and [^18^F]FAZA. On the other hand, [^18^F]HX4 showed the highest variance between patients in both clearance and contrast [47,48]. In sum, each hypoxia tracer has distinct strengths and weaknesses. While [^18^F]FMISO is reported as the most reproducible, albeit with a lower image contrast [48], this is in contrast to [^18^F]FMISO, the first generation of hypoxia imaging agents [49].

**Figure 3:**
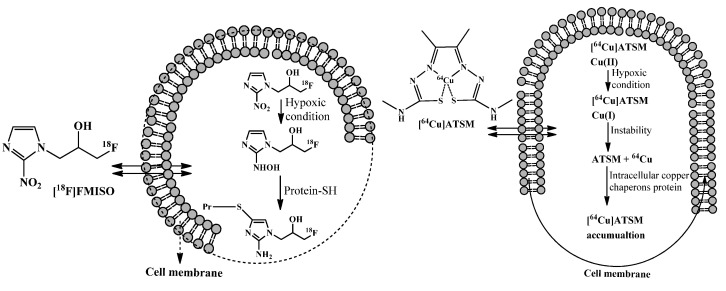
Different radiopharmaceuticals for measuring hypoxia. FMISO is believed to bind covalently to macromolecules in hypoxic cells after reduction of its nitro group [51]. In a hypoxic condition, the Cu(II)-ATSM (oxidation level of copper-64 is +II) is reduced to Cu(I)-ATSM (oxidation level of copper-64 is +I) then the complex becomes unstable and free copper-64 is trapped and accumulated in intracellular copper chaperone proteins [52].

Extensive studies have been performed on [^64^Cu]Cu-Diacetyl-bis(N4-methylthiosemicarbazone) ([^64^Cu]Cu-ATSM), another hypoxia tracer that has numerous advantages over the above mentioned hypoxia tracers. For example, [^64^Cu]Cu-ATSM exhibits more convenient synthetic methods that make radiolabeled ATSM derivatives accessible; a higher hydrophilicity due to the chemical structure of [^64^Cu]Cu-ATSM that makes shorter diagnostic procedures possible as clearance from non-target tissues is faster; and a simpler method for quantification [50]. These factors make [^64^Cu]Cu-ATSM a more favorable tumor hypoxia radiopharmaceutical. Moreover, evidence from clinical studies has shown superior image quality with [^64^Cu]Cu-ATSM relative to [^60^Cu]Cu-ATSM, which is likely due to the unique physical properties of copper-64, leading to a higher signal to noise ratio in obtained images (Figure 3) [50].

Although [^18^F]FDG PET/CT is known as the gold standard diagnostic method in nuclear medicine, there remain some limitations possibly due to a lack of anatomical landmarks or the suboptimal specificity of metabolic imaging [50,51]. One considerable deficiency of [^18^F]FDG is the inability to determine primary tumor volumes as well as assessment of stromal invasion, parametrial involvement, and <1 cm invasion to adjacent organs (vagina, bladder, and rectum) [52,53]. Based on these observations, the development of more specific gynecological radiopharmaceuticals seems necessary.

## 3. Ovarian Cancer

**Figure 4:**
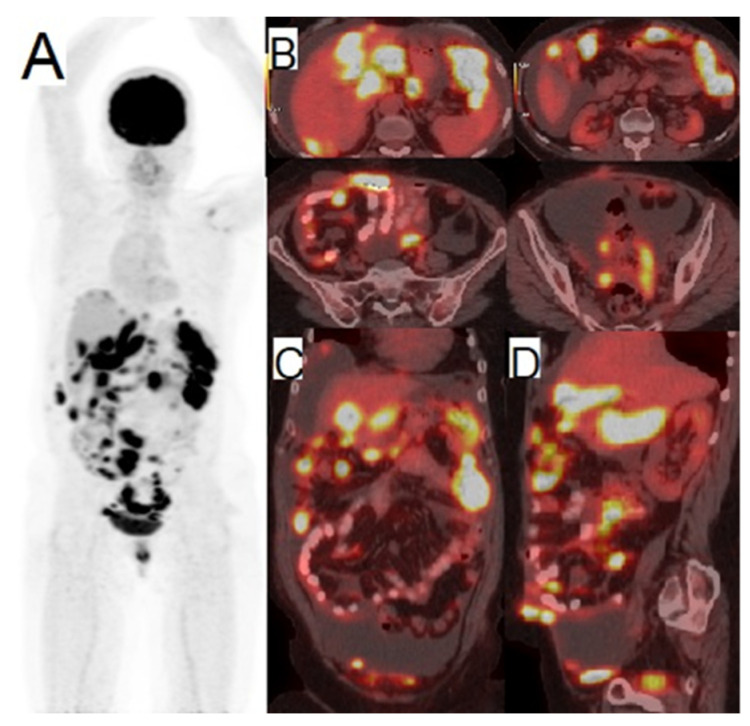
A 50-year-old female with a history of ovarian cancer, seven months post total abdominal hysterectomy with bilateral salpingo-oophorectomy (THA-BSO) with complaints of abdominal pain end measures of elevated tumor markers. (**A**) Anterior maximal intensity projection (MIP) [^18^F]FDG PET/CT image. (**B**) transvers, (**C**) coronal and (**D**) Sagittal abdominopelvic fused PET/CT images showed disseminated intraperitoneal hypermetabolic tumor seeding in abdomen and pelvic cavity as well as hypermetabolic omental thickening (omental cake) and ascites. (Image with courtesy of the Department of Nuclear Medicine, Vali-Asr Hospital, Tehran University of Medical Sciences, Tehran, Iran).

In recent years, ovarian cancer has become the fifth most common cause of death among women all over the world [53]. Early detection of ovarian cancer (stage I) leads to successful treatment in more than 90% of cases. However, this percentage dramatically decreases to 20–25% in later stages (III, IV) [54]. Various diagnostic modalities provide diverse clinical information for the diagnosis of the disease [53]. Molecular imaging modalities including SPECT/CT and PET/CT possess functional information about the biochemistry of tissues [53]. Molecular PET imaging agents reflect general information about energy consumption through glucose metabolism ([^18^F]FDG) or the proliferation of DNA synthesis ([^18^F]F-fluorodeoxythymidine ([^18^F]FLT)) [55]. For more specific targeting of the cell surface, components like hormone receptors, receptor tyrosine kinases, angiogenesis components, and immunotherapy components would be invaluable [55]. Many studies have been conducted to optimize the effective diagnosis and treatment of ovarian cancer. In a comparison study of 51 patients with peritoneal lesions arising from ovarian cancer, it was demonstrated that the obtained visual results of [^18^F]FDG PET/CT in association with other semi-quantitative parameters were effective in the detection of ovarian cancer [56]. This result was based on the observed differentiation potency of [^18^F]FDG PET/CT in malignant and benign lesions [56]. In another study, results showed that CA125 acted as a sensitive tumor marker of recurrent ovarian cancer in 175 patients with recurrent refractory ovarian cancer and increased carcinoma antigen 125 (CA125). Specifically, it was demonstrated that the detection rate of [^18^F]FDG PET/CT scan is 90% for elevated CA125 and 53% for a low (<30) but measurable amount of CA125 [57]. These findings show that [^18^F]FDG PET/CT can detect active lesions despite a low level of CA125, and this can be useful for the early detection and treatment of recurrent cases [57]. Undoubtedly, with increased CA125 (≥35) the diagnostic value of [^18^F]FDG PET/CT has been well established in numerous studies on ovarian cancer [58,59]. Generally, it can be argued that [^18^F]FDG PET/CT is a valuable detection method in suspected recurrent cases, and it acts as a viable prediction tool for the progression of advanced ovarian cancer (Figure 4) [60,61,62,63,64]. Despite these beneficial aspects of [^18^F]FDG PET/CT, however, this procedure doesn’t show reliable diagnostic value in the primary stages of ovarian cancer [55].

### 3.1. DNA Synthesis and Proliferation Imaging Radiopharmaceuticals for Ovarian Cancer

The radiopharmaceutical, [^18^F]FLT, is a tracer for proliferation activity. After internalization, [^18^F]FLT undergoes phosphorylation by thymidine kinase 1, resulting in sequestrated intracellular radioactivity [65]. Thymidine kinase participates in DNA synthesis and therefore reflects the proliferation rate in tissues. Evidence from both preclinical and clinical studies shows a decrease in [^18^F]FLT uptake after ovarian cancer treatment [66,67,68]. Moreover, a pilot study demonstrated that prior to the debulking surgery of ovarian cancer in six patients, [^18^F]FLT showed a higher uptake in tumors compared to normal tissues [68]. Additionally, clinical studies have reported that, due to a high background in liver and bone marrow, administration of [^18^F]FLT for pretreatment assessments would not be recommended given that it may cover the metastases located adjacent to the mentioned organs with a high background [69].

### 3.2. Estrogen Receptor Imaging Radiopharmaceuticals for Ovarian Cancer

Previous studies have shown that in estrogen receptor (ER) positive early breast cancer patients, endocrine therapeutic procedures reduce recurrences and the mortality rate, regardless of whether chemotherapy is also applied [70]. Previous clinical trials have also demonstrated that endocrine therapy for ovarian cancer can improve the response to treatment and prolong survival in platinum-resistant ovarian cancer patients [71,72,73,74]. Based on these findings, ER receptors could be a valuable predictor for patients who may benefit from endocrine hormonal therapy [75].

The 16a-[^18^F]-fluoro-17b-estradiol ([^18^F]FES) PET/CT has been successfully applied in breast and ovarian cancer; and [^18^F]FES uptake has shown a high correlation with estrogen receptor (ER) expressions in previous studies [76,77]. In a clinical study on estrogen-receptor-positive primary breast cancer patients, hormonal therapy failure in [^18^F]FES negative cases was investigated [77]: [^18^F]FES sensitivity and specificity for detection of ER positive lesions was estimated at 84% to 94% for breast cancer and 79% to 100% for ovarian cancer, respectively [53]. Additionally, [^18^F]FES has been demonstrated in leiomyoma as well as epithelial ovarian cancer [77,78]. In 15 patients with suspected ovarian cancer, 88% exhibited lesions measurable with CT and that could be diagnosed with [^18^F]FES PET/CT. The remainder were non-quantifiable due to a high radioactivity uptake of adjacent tissues [78]. These findings support the beneficial role of [^18^F]FES in hormonal therapy.

The tumor antigen mesothelin is also frequently overexpressed in ovarian cancer compared to normal tissues, making it a viable target for the diagnosis or the treatment of ovarian cancer [79]. Previous studies have shown MMOT0530A as an appropriate antibody for mesothelin was radiolabeled with zirconium-89 [80], while phase I clinical trials have shown accumulation of the radiolabeled antibody in both primary and metastatic lesions [80]. Further studies are needed to validate more specific and sensitive tracers, many of which are currently undergoing preclinical trials.

### 3.3. Endometrial Cancer

Endometrial cancer is the most common cancer of the genital tract and the fourth most common malignancy among women in developed countries [81]. Endometrial cancer exhibits a more positive prognosis in that it can often be diagnosed earlier and, for localized occurrences, a five-year survival rate is usually expected (96% of cases) [82]. Nevertheless, overall survival declines to 57% in patients with regional metastasis in pelvic lymph nodes (PLN), and 49.4% in those with metastasis to para-aortic lymph nodes (PALN), with or without positive PLN [83]. Numerous studies have emphasized the unique role of [^18^F]FDG PET/CT in the assessments of staging, restaging, monitoring, and planning of therapeutic procedures in uterine cancers [84,85,86,87]. The reliability of [^18^F]FDG PET/CT in the detection of pelvic and/or para aortic lymph nodes metastasis in patients with untreated endometrial cancer was evaluated in several clinical studies [88,89,90,91], generally showing high efficacy. Furthermore, a meta-analysis [89] also highlighted the utility of [^18^F]FDG PET/CT in the diagnosis of lymph node metastasis (LNM) in pre-operational investigations and post-operative recurrences of endometrial cancers. In order to verify the post-operational effect of [^18^F]FDG, 90 patients with endometrial cancer history were involved in a clinical study designed to investigate residual tumors after curettage [92]. The results support that [^18^F]FDG PET/CT can be used for exact determination of residual tumors in endometrial cancers [92]. Furthermore, it was concluded that in patients with low grade carcinomas and lesion sizes <1.35 cm, [^18^F]FDG uptake would be low, possibly leading to false negative results [92]. In a notable study with coupled [^18^F]FES and [^18^F]FDG PET, it was shown that both approaches are advantageous for the differentiation of malignant and benign uterine tumors [93,94]. It was further demonstrated that the estrogen dependency and the glucose tendency of tumor cells decrease and increase respectively, each correlating with tumor aggression in endometrial carcinomas [94]. Moreover, this observation also highlighted the differences in the [^18^F]FES and the [^18^F]FDG accumulation rates, as related to estrogen expression and glucose consumption [94]. Considering these differences, the [^18^F]FDG–to–[18F]FES ratio may be the most informative index reflecting tumor aggressiveness [94]. Taken together, these findings may assist in developing non-invasive methods for guiding decisions regarding the early detection of and the optimal therapeutic processes for gynecological cancers.

## 4. Vulvar Cancer

Vulvar cancer is a comparatively rare type of neoplasm accounting for 1–5% of the total cancer types in women, and it is more frequent in older women [95]. Distant metastases are very rare in vulvar cancer while lymph node dissemination is observed in 30% of patients [96]. Sentinel node biopsy (SNB) is a gold standard method for staging vulvar cancer without lymphatic spread, and it is useful in preventing postsurgical morbidity [18]. One pervasive issue, however, is the imaging of metastatic LNs. In a clinical trial carried out by Crivellaro et al., 29 patients (mean age 69 years, range 51–88) with vulvar cancer (clinical apparent stage I-II) underwent a pre-operative [^18^F]FDG PET/CT scan [97]. The results showed that [^18^F]FDG PET/CT had low sensitivity and moderate specificity in nodal staging; and, thus, it was not an optimal tool for nodal status assessment. Furthermore, PET/CT may not be cost-effective in detecting the rare event of distant metastases in early stages. Nevertheless, further studies on larger samples are essential to clarify the exact role of [^18^F]FDG PET/CT scans for this purpose.

Finally, ^99m^Tc-labeled colloids have been considered for detection of sentinel node (SLN) using planar scintigraphy and, more recently, SPECT or SPECT/CT [98,99,100,101]. There is evidence from studies in vulvar cancer patients that indocyanine green (ICG)-[^99m^Tc]Tc-nanocolloid SPECT/CT can be used for personalized lymphatic mapping, possibly providing detailed information about the number and the anatomical location of SNs for adequate surgical guidance [98,102,103].

## 5. Vaginal Cancer

Vaginal cancer accounts for approximately 1–2% of gynecological malignancies, among which squamous cell carcinomas and melanoma (less than 4% of vaginal tumors) are the most common [104]. The use of SLN mapping with radiocolloids is beneficial for both diagnosis as well as therapy [105,106]. The most common procedure for detection of LNs is preoperative lymphoscintigraphy using [^99m^Tc]Tc-colloids, following a simultaneous intraoperative blue dye procedure and gamma probe [107]. Clinical trials have shown that, in patients undergoing joint lymphoscintigraphy and blue dye procedures, there was a detection rate of 82% SLN, while just 9% of LNs were detected when using each method separately [108]. In 14 patients with vaginal cancers (including 7 squamous cell carcinomas, 5 vaginal melanomas, 1 adenocarcinoma, and 1 undifferentiated carcinoma), at least one lesion was detectable in 79% of all patients and in each case [108]. Many case reports have also demonstrated that SPECT/CT lymphoscintigraphy is a feasible and an ideal method for pre-operative mapping in vaginal cancer [109,110,111,112,113,114,115,116]. However, in several studies, false negative cases have also been reported [117,118,119].

**Figure 5:**
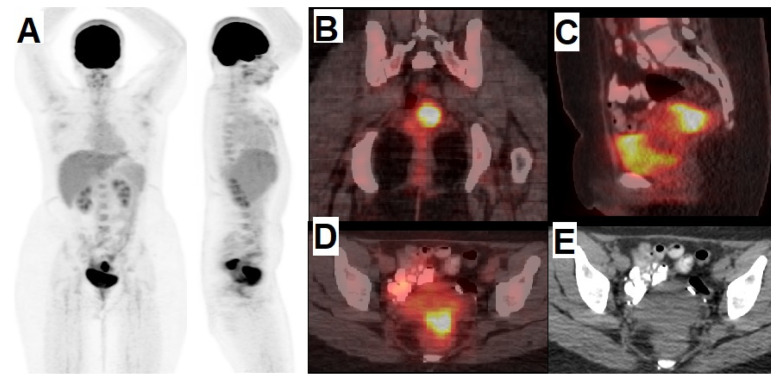
21-year-old female, known case of vaginal rhabdomyosarcoma, referred for staging. (**A**) anterior and right lateral MIP [^18^F]FDG PET/CT images. (**B**) coronal, (**C**) sagittal, (**D**) transvers pelvis fused [^18^F]FDG PET/CT images and (**E**) corresponding transvers pelvis CT image showed hypermetabolic primary tumor without evidence of regional lymphadenopathy or distance metastasis.

In sum, it can be argued that, although SLN detection is not a standard of care to date, efforts are being made to develop non-invasive and effective methods to reduce surgical morbidity without impacting its efficacy in patients affected by vaginal cancers. Further studies are needed, however, to confirm the reliability and the accuracy of SLN mapping by blue dye as well as radiopharmaceuticals in gynecologic oncology. However [^18^F]FDG PET/CT could be still a gold standard for imaging purpose of vaginal malignancies (Figure 5) [120,121,122].

## 6. Recent Advances of [^68^Ga]Ga-FAPI in Various Gynecological Cancers

The current and most frequently used PET/CT diagnostic radiotracer ([^18^F]FDG) in oncology, accumulates based on glucose consumption. Therefore, [^18^F]FDG uptake is influenced by glucose level, physical movement, and nutrition [123,124]. However, there are considerable limitations to this method including high physiological background activity, low glucose transporter density, and varying hexokinase activity in some malignant tissues, which can lead to a decreased specificity when making a diagnosis [124,125]. The radiolabeled fibroblast activation protein inhibitor (FAPI) is a novel class of radiopharmaceuticals that has shown promising diagnostic results for various tumor types [126,127]. A type II serine protease, the fibroblast activation protein (FAP) is expressed by cancer associated fibroblasts (CAFs), and CAFs are associated with stroma in many tumors with poor prognosis [128,129,130]. With respect to the limitations of [^18^F]FDG, it can be argued that radiolabeled FAPIs for PET/CT diagnosis would be superior in gynecological cancers and also in many malignant and non-malignant tissues [131]. In a cohort study, 31 patients (median age 59.5) from two centers with several gynecological tumors (breast cancer; ovarian cancer; cervical cancer; endometrial cancer; leiomyosarcoma of the uterus; tubal cancer) underwent [^68^Ga]Ga-FAPI PET/CT [131]. In 8 patients, primary tumors were detectable, and in all 31 patients metastases were identified. Notable outcomes resulted from a comparison between the biodistribution of [^18^F]FDG and [^68^Ga]Ga-FAPI in normal organs. The results showed that mean SUVmax of FAPI was significantly lower in most normal organs [131]. Mean SUVmax showed a statistically significantly lower uptake for [^68^Ga]Ga-FAPI compared to [^18^F]FDG in brain parenchyma ([^68^Ga]Ga-FAPI vs. [^18^F]FDG: 0.1 vs. 10.8; *p* = 0.005), oral mucosa (1.9 vs. 2.8; *p* = 0.028), parotid gland (1.4 vs. 2.0; *p* = 0.044), myocardium (1.5 vs. 3.2; *p* = 0.017), blood-pool (mean SUVmax 1.8 vs. 2.3; *p* = 0.009), liver (1.3 vs. 3.0; *p* = 0.005), pancreas (1.4 vs. 2.0; *p* = 0.021), spleen (1.4 vs. 2.5; *p* = 0.012), kidney cortex (2.1 vs. 2.7; *p* = 0.007), gastrointestinal tract (measured in colon transversum: 1.3 vs. 2.0; *p* = 0.008), spinal canal (0.7 vs. 1.0; *p* = 0.028), and bone tissue (1.1 vs. 2.3; *p* = 0.028) [131].

Moreover, ^68^Ga-FAPI PET/CT scans can be accomplished with no requirement for patients to rest or to fast, nor are they affected by blood glucose level. This procedure can also be completed comparatively quickly, with lower off-target accumulation relative to [^18^F]FDG [132,133]. In a clinical study by Wang et al., the comparison of diagnostic results achieved through [^18^F]FDG and [^68^Ga]Ga-FAPI-04 showed that physiological accumulation of [^68^Ga]Ga-FAPI-04 in the ovaries is not affected by the menstrual cycle. This finding was in contrast to the reported effects of [^18^F]FDG, which exhibited accumulation in both malignant and functional ovarian lesions. This is a clear indicator of [^68^Ga]Ga-FAPI as a differentiation radiotracer in gynecological malignancies [134].

Notably, radiolabeled FAPI may also be used as a theranostic tracer, which is highly promising for staging, re-staging, and follow-up of gynecological malignancies. However large prospective studies are needed to gain more information about the specificity, sensitivity, and accuracy of [^68^Ga]Ga-FAPI PET/CT in gynecological cancers [131].

## 7. Conclusions

In this review, we have provided a detailed summary of various radiopharmaceuticals that are used to assist in the accurate diagnosis of gynecological malignancies. However, these concepts can also be extended to other oncological conditions. Nuclear medicine, in combination with radiological modalities, gives extra information for diagnosis, prognosis, staging, treatment management, and the evaluation of responses to therapy in a non-invasive manner. Based on these descriptions, nuclear medicine plays a key role in the clinical evaluation of oncological malignancies. Given the extraordinary effects of gynecological cancers on female health worldwide, the need for the development of more specific radiopharmaceuticals is absolutely essential. Finally, though the use of these techniques in gynecological and obstetric cancers is valuable, it should also be noted that their availability may be currently limited. For example, while the use of Cu-64 ATSM has been approved by the United States Food and Drug Administration (and are therefore the only radiopharmaceuticals next to FDG which are widely available for patients in the US), availability of this and other such radiopharmaceuticals will vary across different countries or territories and the local regulatory framework. As a result, further clinical studies are critical to quantify and to determine the exact potential of these radiopharmaceuticals in the diagnosis and the treatment of gynecological and obstetric malignancies.
